# A tumor microenvironment-activated metal-organic framework–based nanoplatform for amplified oxidative stress–induced enhanced chemotherapy

**DOI:** 10.1016/j.jbc.2022.102742

**Published:** 2022-11-23

**Authors:** Bo Li, Xin Yao, Jiaqi Li, Xin Lu, Wen Zhang, Wenyao Duan, Yupeng Tian, Dandan Li

**Affiliations:** 1 Institutes of Physics Science and Information Technology, Key Laboratory of Structure and Functional Regulation of Hybrid Materials, Ministry of Education, Anhui University, Hefei, China; 2 Department of Chemistry, Key Laboratory of Functional Inorganic Material Chemistry of Anhui Province, Anhui University, Hefei, China

**Keywords:** metal-organic framework, chemotherapy, disulfiram, amplified oxidative stress, tumor microenvironments, AA, ascorbic acid, DMF, N,N-dimethylformamide, DSF, disulfiram, FA, folic acid, MOF, metal-organic framework, TME, tumor microenvironment

## Abstract

Engineering a highly tumor microenvironment-responsive nanoplatform toward effective chemotherapy has always been a challenge in targeted cancer treatment. Metal-organic frameworks are a promising delivery system to reformulate previously approved drugs for enhanced chemotherapy, such as disulfiram (DSF). Herein, a tumor microenvironment-activated metal-organic framework–based nanoplatform **DSF@MOF-199@FA** has been fabricated to realize amplified oxidative stress–induced enhanced chemotherapy. Our results unveil that the copper ions and DSF released by **DSF@MOF-199@FA** in an acidic environment can be converted into toxic bis(N, N-diethyl dithiocarbamate) copper and then induce cell apoptosis. Simultaneously, we determined that the apoptosis outcome is further promoted by amplified oxidative stress through effective generation of reactive oxygen species and GSH elimination. In conclusion, this work provides a promising platform for effective anticancer treatment.

Chemotherapy still leads to the most prevalent modalities among a variety of anticancer treatments in recent years, although all kinds of treatment methods have developed (1, 2, 3, 4). Currently, commonly used chemotherapy drugs include doxorubicin (5), cisplatin (6), paclitaxel (7), tirapazamine (8), platinum(Ⅳ) (9), camptothecin (10) and so on. In addition, as an old drug approved by the U.S. Food and Drug Administration (FDA), disulfiram (DSF) has been used for treating alcohol dependence for over 6 decades. Recent research illustrated that it can turn into toxic bis(N, N-diethyl dithiocarbamate) copper (II) (CuET) after chelated with Cu(II) to induce heat shock response and cancer cell death (11, 12, 13, 14). However, the serious side effect, high system toxicity, and unsatisfactory therapeutic efficacy limited its application (15). Considering the high cost from the research of the pharmacokinetics and safety profiles of new drugs, searching for new anticancer drug formulations based on DSF is an attractive strategy to combat the aforementioned issue.

Metal-organic frameworks (MOFs), consisting of metal nodes and organic ligand, have been widely used in various advanced fields, such as gas sorption and separation, catalysis, food safety, drug delivery, and cancer therapy (16, 17, 18, 19, 20, 21). In particular, MOFs recommended themselves as very promising hosts for old drugs loading to develop new anticancer drug formulations due to their high porosity (22, 23, 24). Of note, their metal nodes offer ample possibilities for fabricating tumor microenvironment (TME)–responsive platforms. For example, some Cu-based MOFs endow their particular merits toward TME-responsive therapy by releasing Cu(II) ions in acid TME. The resultant Cu(II) could trigger GSH depletion (25, 26) and Cu(I)-mediated ∙OH generation *via* self-cyclic valence alternation (27, 28), giving rise to amplified oxidative stress and further improve the chemotherapeutic effect (29, 30). In this sense, Cu(II)-based MOFs hold great advantages in promoting the CuET-mediated chemotherapeutic by the amplified intracellular oxidative stress.

Bearing the aforementioned considerations in mind, we employed the acid-responsive **MOF-199** as the main material to fabricate an intelligent delivery system (**DSF@MOF-199@FA**) to promote the chemotherapy effect. As illustrated in Figure 1, the folic acid (FA) was wrapped on the surface of **DSF@MOF-199** to endow it (**DSF@MOF-199@FA**) with cancer cell–specific targeting ability (31). In addition, the TME-responsive system could release Cu(II) ions and DSF. Thereinto, DSF could convert into CuET (Figs. S1 and S2) *in situ* to induce cell apoptosis. Besides, the released Cu(II) ions could be used to amplify intracellular oxidative stress by consuming intracellular GSH and generating ∙OH (generated from the Fenton-like reaction) *via* self-cyclic valence alternation. Finally, this **DSF@MOF-199@FA** mediated amplified oxidative stress strategy provides a new paradigm to amplify the CuET chemotherapeutic effect.

**Figure 1:**
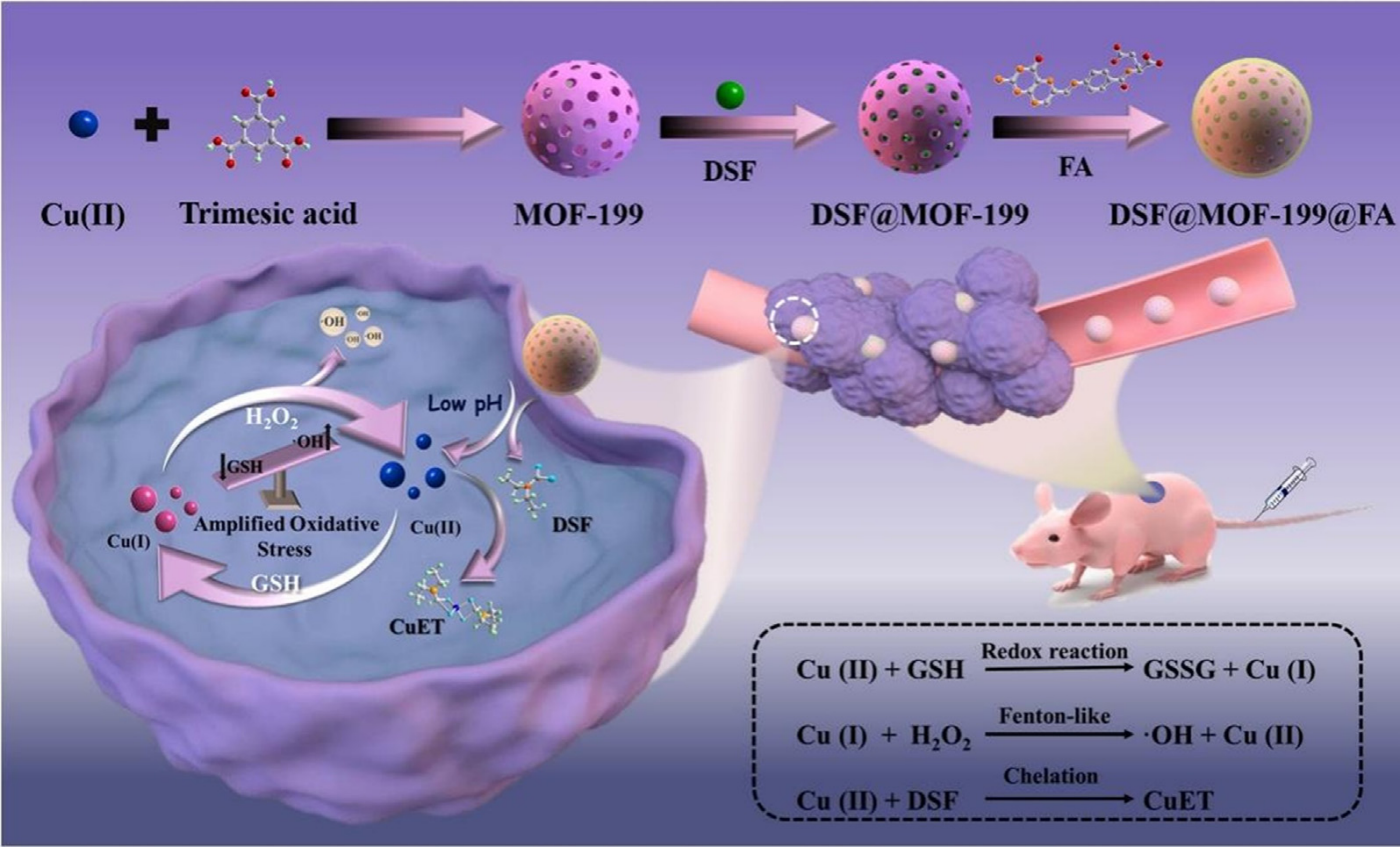
**Scheme illustration showing the preparation ofDSF@MOF-199@FA, highlighting the amplified oxidative stress enhancedCuET-mediatedchemotherapy.** DSF, disulfiram; FA, folic acid; MOF, metal-organic framework.

## Results and discussion

**Figure 2:**
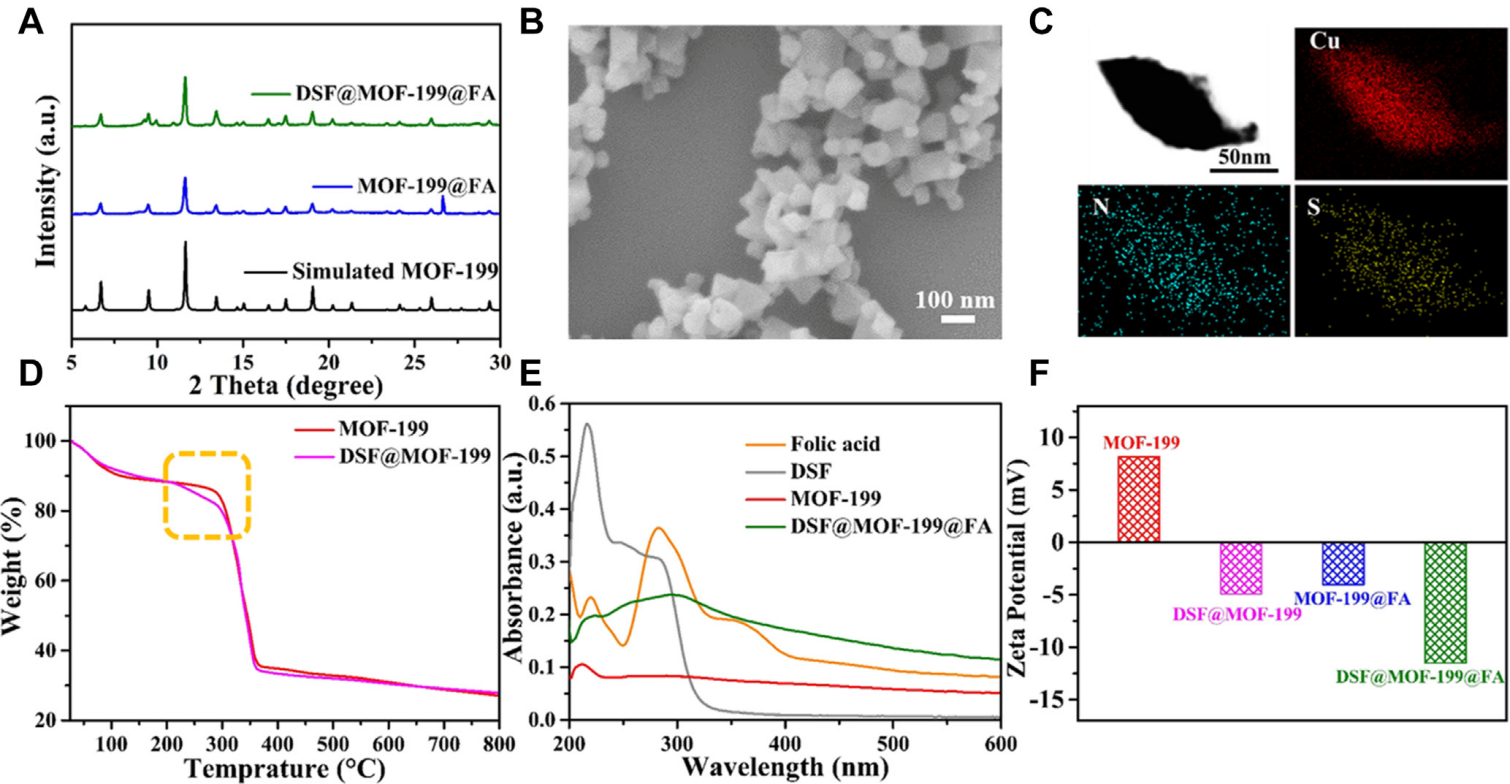
**Characterization of DSF@MOF-199@FA.***A*, PXRD patterns of simulated **MOF-199**, **MOF-199@FA**, and **DSF@MOF-199@FA**. *B*, scanning electron microscopy image of **DSF@MOF-199@FA**. *C*, elemental mapping images of the ultrathin slice of **DSF@MOF-199@FA** in TEM. *D*, TGA curves of **MOF-199** and **DSF@MOF-199**. *E*, UV-vis absorbance spectra of folic acid, DSF, **MOF-199**, **DSF@MOF-199@FA**. *F*, zeta potentials of **MOF-199**, **DSF@MOF-199**, **MOF-199@FA**, and **DSF@MOF-199@FA**. DSF, disulfiram; FA, folic acid; MOF, metal-organic framework; PXRD, power X-ray diffraction.

**DSF@MOF-199@FA** was fabricated by loading DSF into the pores of defective **MOF-199** through simply physical absorption and then wrapped with FA. As identified by Figure 2*A*, the power X-ray diffraction pattern of **MOF-199** showed the same characteristic peaks as the simulated one, illustrating the **MOF-199** was successful synthesized. The crystal structure and crystallinity of **DSF@MOF-199**, **MOF-199@FA**, and **DSF@MOF-199@FA** remained well compared with **MOF-199**. Compared with **MOF-199**, the obvious diffraction peaks at 2*θ* = 9.25°, 9.95° in **DSF@MOF-199** and **DSF@MOF-199@FA**. Besides, in terms of **DSF@MOF-199** and **DSF@MOF-199@FA**, the emerged small diffraction peaks (2θ = 9.25°, 9.95°) could be attributed to the DSF loading given the interaction between DSF molecules and Cu ions of MOFs, which was revealed by power X-ray diffraction and X-ray photoelectron spectroscopy measurements (Figs. S3 and S4). The scanning electron microscopy image in Figure 2*B* and transmission electron microscopy image in Fig. S5 of **DSF@MOF-199@FA** displayed the unchanged octahedron morphology, suggesting the absence of structure and morphology variation of **MOF-199** after loading with DSF and coating with FA.

Besides, the **DSF@MOF-199@FA** was sliced into ultrathin slices and the elemental mapping images were collected, in which Cu was attributed to **MOF-199**, S was assigned to DSF, and N contributed to DSF and FA (Fig. 2*C*), which demonstrated that the DSF was loaded into the pores of **MOF-199**. As shown in Figure 2*D*, the weight loss between 200 °C and 300 °C was attributed to the DSF decompose (32), which further demonstrated that DSF was efficiently loaded into **MOF-199**. The loading efficiency of DSF was calculated to be 4.9% in the **DSF@MOF-199@FA** by inductively coupled plasma atomic emission spectrometry measurement. The UV-visible absorption spectrum of **DSF@MOF-199@FA** displayed significant change after subsequent DSF loading and FA coating, a new absorption band appeared (230–350 nm) due to the DSF and FA absorption (Fig. 2*E*). Particularly, the absorption band around 260 to 320 nm showed a difference between **DSF@MOF-199** and **DSF@MOF-199@FA**, which was attributed to the FA coating. Besides, the new peak around 1608 cm^−1^ in FTIR spectrum increased after modified by FA was contributed to the -NH stretching vibrations of -NH_2_ in FA (Fig. S6). In addition, as displayed in Figure 2*F*, the zeta potential experiment indicated a change in the surface charge from a positive potential for **MOF-199** (+8.04 mV) to a negative potential for **MOF-199@FA** (−4.05 mV) and **DSF@MOF-199** (−4.92 mV) after FA modification and DSF loading, respectively. The potential was further reduced to −11.5 mV (**DSF@MOF-199@FA**) after loading with DSF and modifying with FA simultaneously. All the aforementioned results demonstrate the successful fabrication of **DSF@MOF-199@FA**.

**Figure 3:**
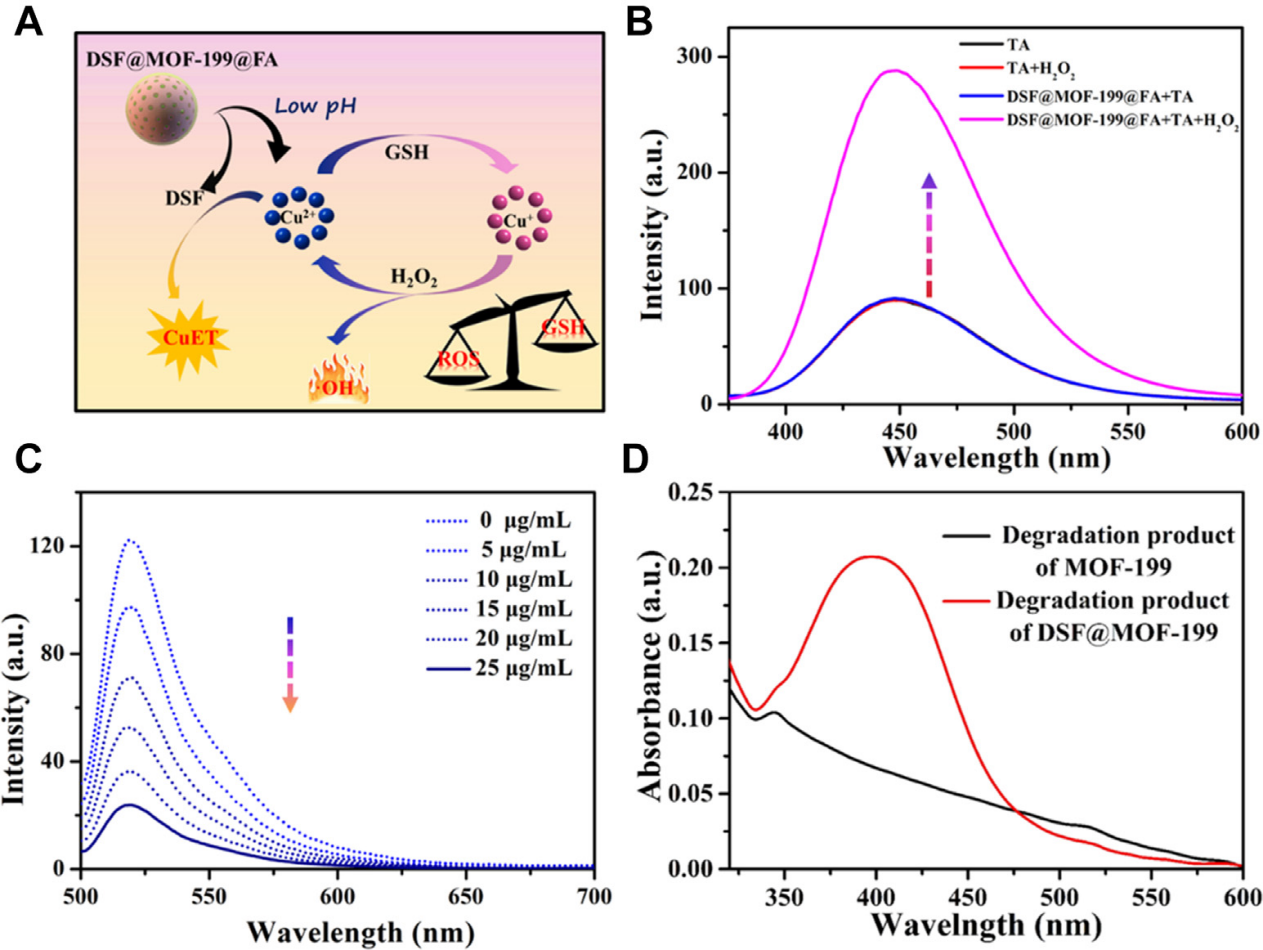
**GSH consumption and Fenton-like reactions of DSF@MOF1-99@FA.***A*, illustration of ROS generation, GSH depleting, and CuET formation process within **DSF@MOF-199@FA**. *B*, determination of the formation of ∙OH treated with **DSF@MOF-199@FA** by terephthalic acid as the fluorescent probe. Reaction conditions: **DSF@MOF-199@FA** (50 μg ml^−1^), TA (0.05 mM), pH = 6.5. *C*, GSH depleting ability of **DSF@MOF-199@FA** at different concentration with thiolite green as the detection agent for GSH. *D*, UV-vis spectrum of degradation product of **MOF-199@FA** and **DSF@MOF-199@FA**. DSF, disulfiram; FA, folic acid; MOF, metal-organic framework; ROS, reactive oxygen species.

Motivated by the successful fabrication of **DSF@MOF-199@FA**, the ROS generation, GSH depleting, and CuET formation performance were studied thoroughly by the *in vitro* experiments. Firstly, the acidity-responsive degradation performance of **MOF-199** was evaluated by scanning electron microscopy observation. As shown in Figs. S7 and S8, the **MOF-199** was physiologically stable after incubating with PBS in neutral condition (pH = 7.4) for 24 h, while degraded quickly in the acidic PBS solution (pH = 6.5), suggesting that the **MOF-199** could degrade and release the copper ions and DSF for anticancer treatment in the acid environment. Furthermore, the polydispersity index of **DSF@MOF-199@FA** maintained stable in serum over 7 days, which confirmed its good stability in the blood circulation (Fig. S9). Then, terephthalic acid, which can react with ∙OH radicals to form 2-hydroxyterephthalic acid was used to evaluate the ∙OH (origin from the Fenton-like reaction) generation capability of **DSF@MOF-199@FA**. With the prolonged incubation time, a fluorescence enhancement around 450 ± 20 nm appeared, indicating that **DSF@MOF-199@FA** is capable of generating ∙OH in the acidic TME efficiently (Figs. 3*B* and S10) while the **DSF@MOF-199@FA** or H_2_O_2_ alone do not generate any ∙OH. The Fenton-like effect of **DSF@MOF-199@FA** was proved to be derived from free Cu^2+^ ions by the control experiments of CuET and Cu^2+^ (Fig. S11). Moreover, thiolite green was selected as the detection agent for GSH. As shown in Fig. S12, the solution showed the faint green fluorescence at 520 nm in the presence of H_2_O_2_ and **DSF@MOF-199@FA**, which proved that **DSF@MOF-199@FA** displayed excellent GSH consumption capacity (Fig. S12). Besides, the fluorescence was gradually faded (Fig. 3*C*) with the concentration of **DSF@MOF-199@FA** increased. In addition, compared with **MOF-199@FA**, the UV-visible spectrum of degradation product originating from **DSF@MOF-199@FA** presented a characteristic peak around 450 nm (the peak of CuET), suggesting that the **DSF@MOF-199@FA** could release copper ions and DSF in the acidic environment and then form CuET complex *in situ* for chemotherapy (Fig. 3*D*). With the extension of incubation time, the absorption at 450 ± 20 nm increased gradually with the extension of incubation time in the acidic environment, which proved the formation of CuET (Figs. S13 and S14). Given **DSF@MOF-199@FA** could generate ∙OH and consume GSH *via* self-cyclic valence alternation, it is implied that **DSF@MOF-199@FA** can promote the CuET-mediated chemotherapeutic effect by amplifying the intracellular oxidative stress.

**Figure 4:**
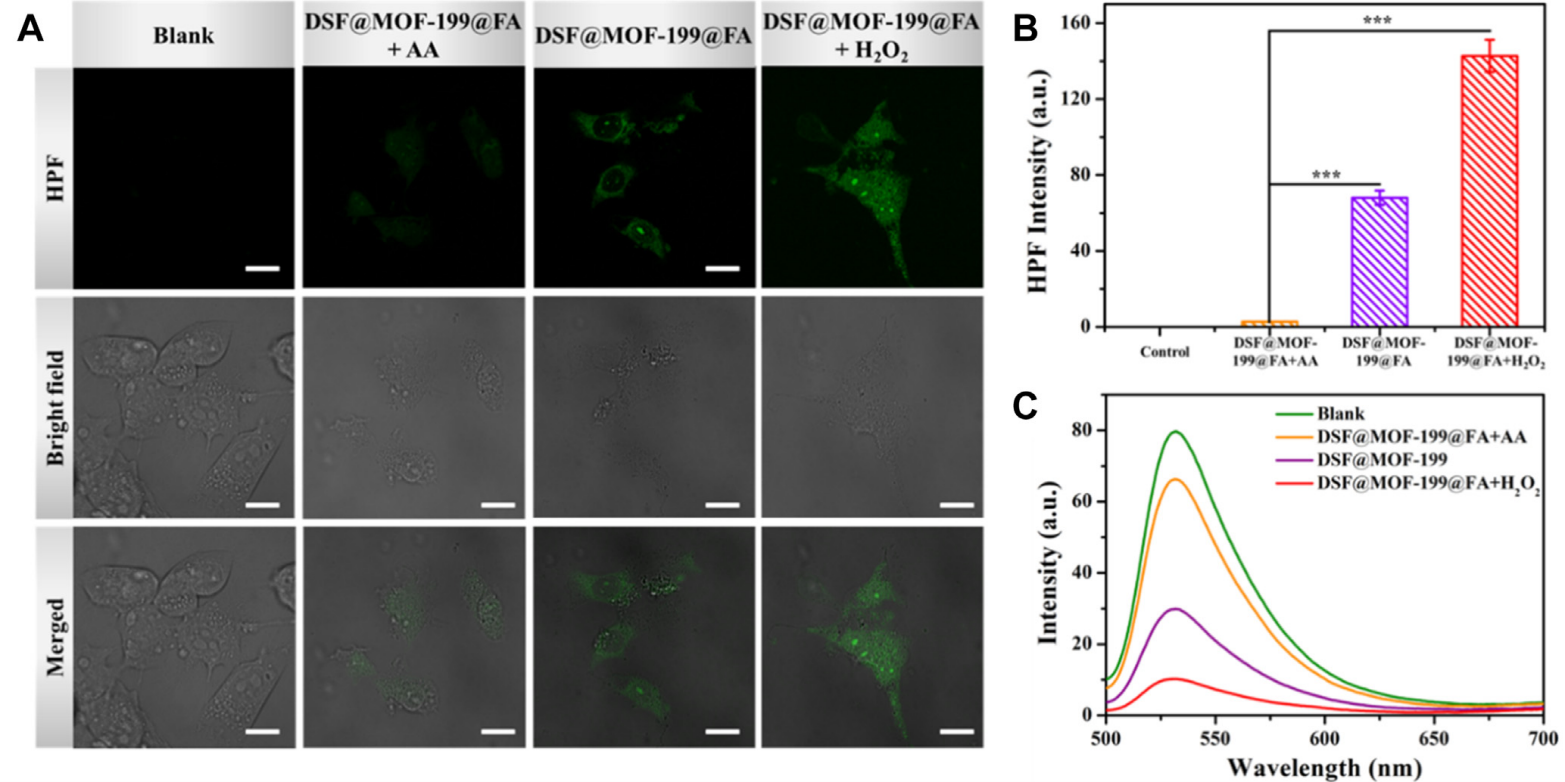
***In vitro* GSH consumption and ∙OH production of DSF@MOF-199@FA GSH consumption and ∙OH production of DSF@MOF-199@FA under different conditions.***A*, intracellular ∙OH evaluation. CLSM fluorescence images of HPF-stained 4T1 cells treated with PBS, **DSF@MOF-199@FA** + AA, **DSF@MOF-199@FA**, **DSF@MOF-199@FA** + H_2_O_2_, reaction conditions: **DSF@MOF-199@FA** (50 μg ml^−1^), H_2_O_2_ (100 μM), AA (25 μg mL^−1^), scale bar = 20 μm. *B*, HPF intensity in 4T1 cells after different treatments. *C*, intracellular GSH levels in 4T1 cells after different treatments. Data are presented as mean ± SD; n.s.: not significant; ∗∗*p* < 0.01, ∗∗∗*p* < 0.001. DSF, disulfiram; FA, folic acid; HPF, hydroxyphenyl fluorescein; MOF, metal-organic framework.

Encouraged by the aforementioned experiments, we decided to investigate the ∙OH generation performance of **DSF@MOF-199@FA** in cancer cells *via* confocal laser scanning microscopy imaging, in which the 4T1 cells were stained with hydroxyphenyl fluorescein. As illustrated in Figure 4*A*, compared with the blank group, the green fluorescence of hydroxyphenyl fluorescein increased significantly for **DSF@MOF-199@FA** with/without H_2_O_2_ groups. Besides, the fluorescence almost disappeared upon ∙OH scavenger (ascorbic acid, AA) added, indicating the ∙OH generation ability of **DSF@MOF-199@FA** within 4T1 cells. Meanwhile, the enhanced fluorescence intensity of **DSF@MOF-199@FA** (Fig. 4*B*) with the addition of H_2_O_2_ demonstrating that the additional H_2_O_2_ is able to facilitate the Fenton-like reaction and further improve ∙OH generation.

Apart from reactive oxygen species, GSH also plays an important role in amplifying oxidative stress. Therefore, thiolite green was utilized to assess the GSH level in the 4T1 cells after different treatments. Obviously, in contrast to the blank group, a decreased GSH concentration could be observed upon **DSF@MOF-199@FA** treated. As displayed in Figure 4*C*, compared with the **DSF@MOF-199@FA** group, the concentration of GSH increased with AA addition, which probably attributed to the inhibition of GSH consumption by AA. Moreover, GSH level could even be reduced further when H_2_O_2_ added. It demonstrated that **DSF@MOF-199@FA** can effectively consume the intracellular GSH due to the released Cu(II) that was reduced to Cu(I) by GSH through redox reaction. The aforementioned results further confirmed that **DSF@MOF-199@FA** can be used to amplify oxidative stress by producing ∙OH and consuming GSH for enhanced chemotherapy.

**Figure 5:**
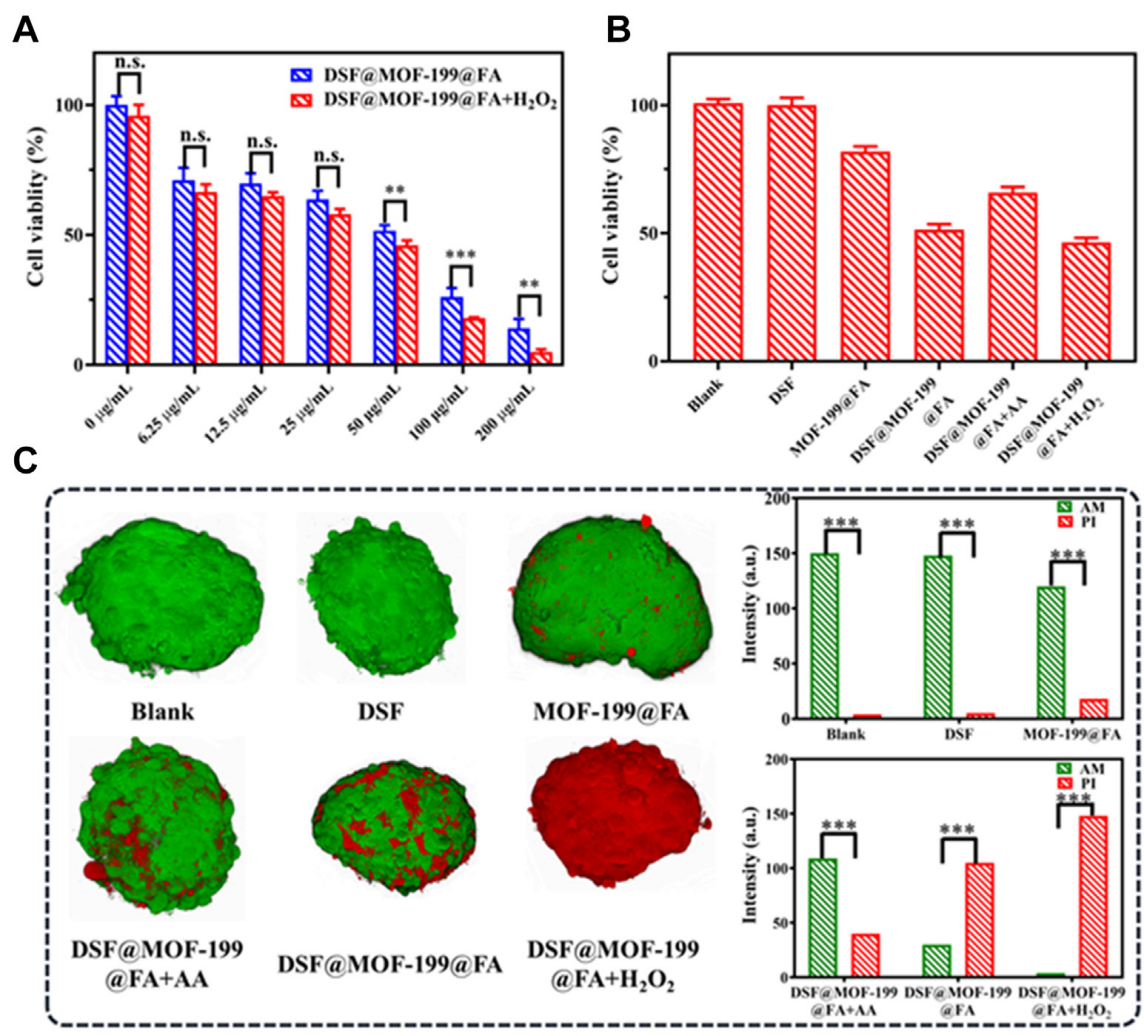
**Analysis of *in vitro* cytotoxicity under different conditions.***A*, cell viability in 4T1 cells after 12 h of incubation with different treatments. *B*, cell viability after varied treatments, including blank, DSF only, **MOF-199@FA**, **DSF@MOF-199@FA** + AA, **DSF@MOF-199@FA**, and **DSF@MOF-199@FA** + H_2_O_2_ (**DSF@MOF-199@FA** (50 μg ml^−1^), **MOF-199@FA** (47.5 μg mL^−1^), DSF (2.5 μg mL^−1^), H_2_O_2_ (100 μM), AA (25 μM)). *C*, 3D fluorescence images of MCTs after different treatments. 4T1 MCTs incubated with AM (indicator of living cells)/PI (indicator of dead cells). Data are presented as mean ± SD; n.s.: not significant; ∗∗*p* < 0.01, ∗∗∗*p* < 0.001. AA, ascorbic acid; DSF, disulfiram; FA, folic acid; MCT, multicellular tumor spheroid; MOF, metal-organic framework; PI, propidium iodide.

To avoid side effects on normal cells, we modified **DSF@MOF-199** with FA to enhance its cancer cell–specific targeting capability. The cancer cell–specific targeting behavior of **DSF@MOF-199@FA** were evaluated by cell uptake experiments with FAR (folate receptor) abundant cells (HeLa: human cervical cancer cell) and FAR negative cells (HEK 293FT: human embryonic kidney cells). To characterize the process of the endocytosis of **DSF@MOF-199@FA** into cancer cells clearly, **DSF@MOF-199@FA** were labeled with FITC (**DSF@MOF-199@FA-FITC**) (Fig. S15). As illustrated in Figs. S16 and S17, **DSF@MOF-199@FA-FITC** preferentially accumulated in HeLa cells but not in HEK 293T cells, HeLa cells which incubated with **DSF@MOF-199-FITC** and the HeLa cells which were incubated with free FA in advance. Then, cell uptake experiments with 4T1 cells (mouse breast cancer cells, FAR abundant cells) also demonstrated that FA-modified can effectively target FAR-overexpressing cancer cells (Fig. S18). Moreover, the cytotoxicity was evaluated *via* standard (4,5-dimethylthiazol-2-yl)-2,5-diphenyltetrazolium bromide assay using HEK 293T cells (human embryonic kidney cell), SW480 cells (human colorectal carcinoma cell), HeLa cells (human cervical cancer cell), and 4T1 cells (mouse breast cancer cell). As shown in Fig. S19, more than 90% of HEK 293T cells survived after being incubated with different concentration of **DSF@MOF-199@FA** (0–80 μg ml^−1^) for 12 h, suggesting their low cytotoxicity to healthy cells and excellent biocompatibility. In contrast, cell viability of 4T1 cells decreased along with an increase in the concentration of **DSF@MOF-199@FA** (Fig. 5*A*), the treated HeLa cells and SW480 cells showed similar results (Fig. S20). The cell viability of 4T1 cells was further decreased after H_2_O_2_ addition because of the amplified oxidative stress trigged by H_2_O_2_. Notably, as shown in Fig. S21, H_2_O_2_ (100 μM) displayed ignorable cytotoxicity. These results reveal that **DSF@MOF-199@FA** can selectively induce cancer cells apoptosis and then avoid side effects effectively.

Strategically, we deployed a systematic protocol to evaluate toxicity of DSF after chelated with Cu(II) ions as well as amplified oxidative stress–induced chemotherapy effect, including standard (4,5-dimethylthiazol-2-yl)-2,5-diphenyltetrazolium bromide and 3D multicellular tumor spheroid assays (Fig. 5). Evidently, in contrast to the ignorable cytotoxicity toward 4T1 cells and 3D multicellular tumor spheroids treated with DSF or **MOF-199@FA**, **DSF@MOF-199@FA** (with equal concentration of DSF) displayed obviously cytotoxicity elaborating the *in situ* generated CuET could serve as a chemotherapeutic agent. **DSF@MOF-199@FA** with H_2_O_2_ treatment group displayed much lower cell viability due to the generated OH originating from the Fenton-like reaction amplified by the intracellular oxidative stress. In addition, upon AA (reduced oxidative stress) added, the survival rates increased.

**Figure 6:**
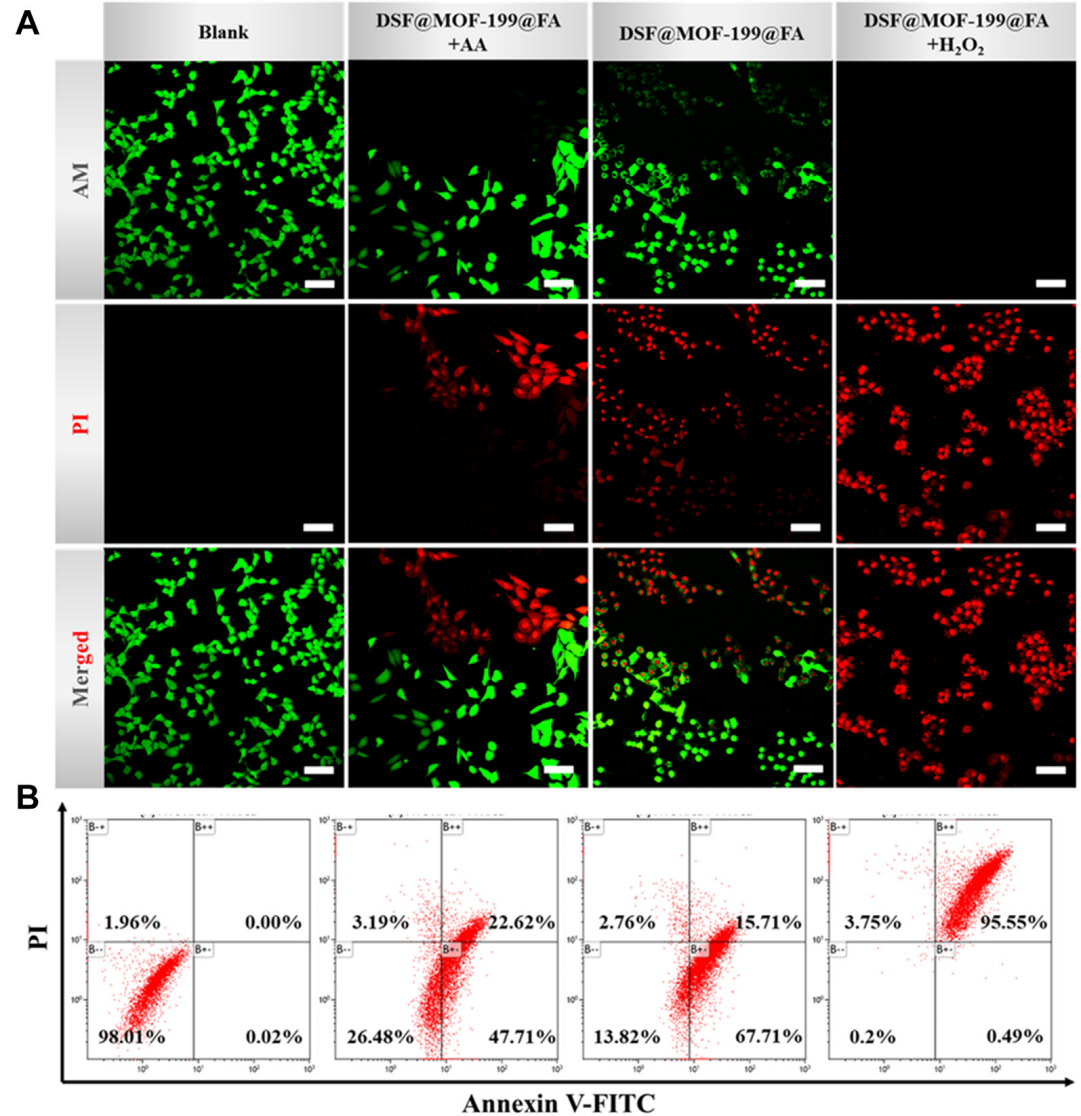
***In vitro* therapy performance of DSF@MOF-199@FA.***A*, CLSM images of 4T1 cells stained with calcein AM/PI after different treatments (scale bar: 100 μm). *B*, 4T1 cells treated with **DSF@MOF-199@FA** apoptosis analyzed by flow cytometry after different treatment using annexin V-FITC and PI as indicators of apoptosis. CLSM, confocal laser scanning microscopy; DSF, disulfiram; FA, folic acid; MOF, metal-organic framework; PI, propidium iodide.

Inspired by the amplified oxidative stress and CuET-mediated chemotherapy of **DSF@MOF-199@FA**, the systematic performance of therapeutic effect was further evaluated by confocal laser scanning microscopy observation (Fig. 6*A*). Red propidium iodide signal could be observed in the **DSF@MOF-199@FA** group demonstrating its chemotherapy outcome against tumor cells. Upon AA (reduce oxidative stress) or H_2_O_2_ (amplify oxidative stress) added, weakened or enhanced propidium iodide signal could be collected, respectively, corroborating the amplified oxidative stress–induced enhanced chemotherapy performance. Moreover, the aforementioned results were further confirmed by flow cytometry (Fig. 6*B*). With the addition of H_2_O_2_, **DSF@MOF-199@FA** induced 99.43% apoptotic cells, which was obviously higher than that of with AA (82.18%) or without H_2_O_2_ (97.05%) treatment. All the aforementioned *in vitro* results showed that **DSF@MOF-199@FA** can be used to enhance CuET-mediated chemotherapy by the amplified oxidative stress.

**Figure 7:**
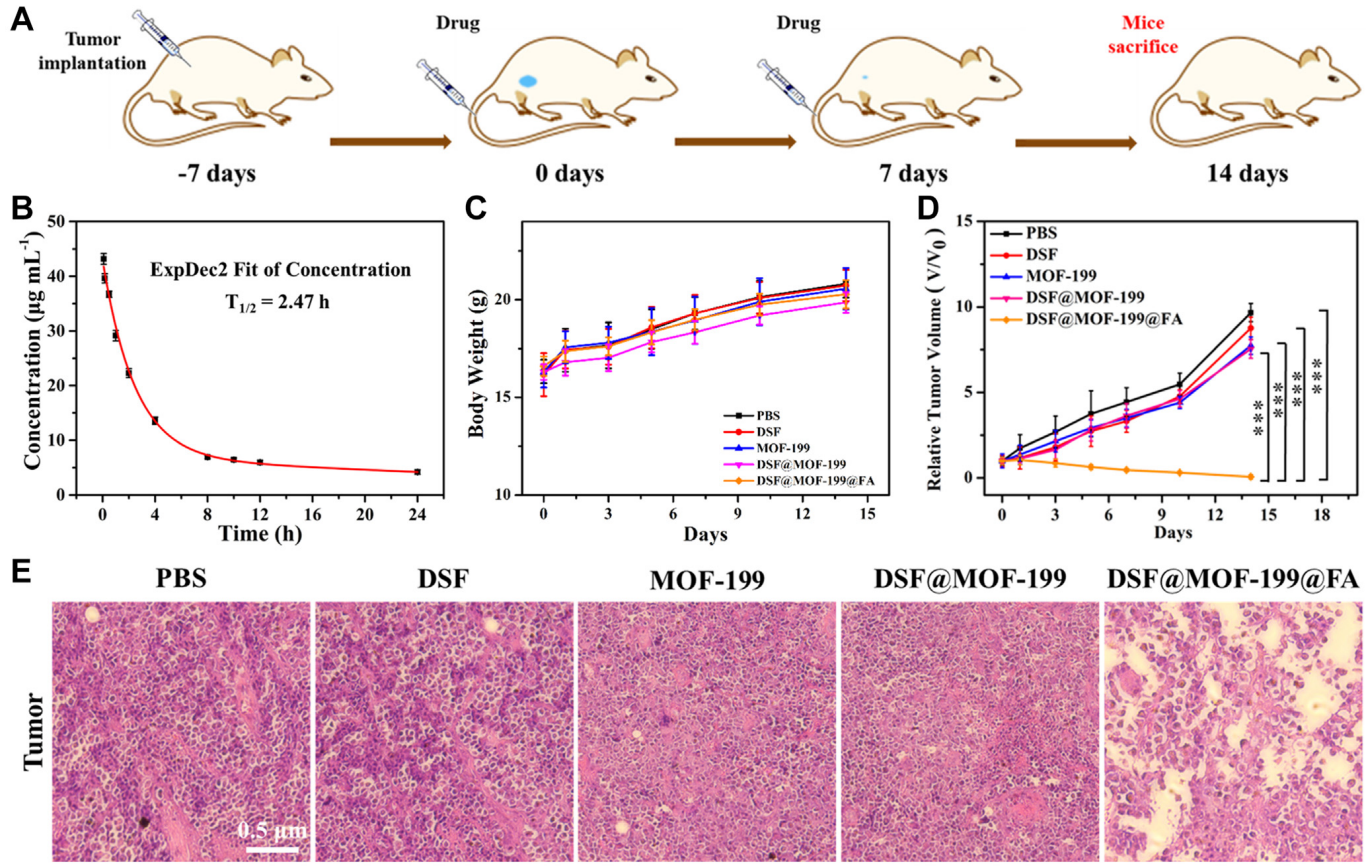
***In vivo* therapy performance of DSF@MOF-199@FA.***A*, schematic diagram of 4T1 subcutaneous tumor-bearing female BALB/C mice model development and treatment process. *B*, blood circulation lifetime of **DSF@MOF-199@FA** after intravenous injection. *C*, curves of body weights of nude mice in various treatment groups (PBS, DSF, **MOF-199**, **DSF@MOF-199**, and **DSF@MOF-199@FA** (n = 5)). *D*, curves of tumor volumes of the PBS, DSF, **MOF-199**, **DSF@MOF-199**, and **DSF@MOF-199@FA** groups (n = 5). *E*, H&E stained sections of tumors in different treatment groups. Data are presented as mean ± SD; n.s.: not significant; ∗∗*p* < 0.01, ∗∗∗*p* < 0.001. DSF, disulfiram; FA, folic acid; MOF, metal-organic framework.

Based on the surprising *in vitro* therapeutic effect of amplified oxidative stress–induced enhanced CuET-mediated chemotherapy, the *in vivo* biological behavior of **DSF@MOF-199@FA**, including circulation, biocompatibility, and anticancer ability were investigated on the 4T1 breast tumor–bearing female BALB/C nude mice. Initially, the pharmacokinetic behavior of **DSF@MOF-199@FA** in blood circulation was studied by intravenous injection, and the half-life was calculated to be 2.47 h within the bloodstream (Fig. 7*B*). Afterward, the female BALB/C mice bearing 4T1 tumor were randomly divided into five groups (n = 5) and intravenous injected with PBS, DSF only, **MOF-199** only, **DSF@MOF-199** only, and **DSF@MOF-199@FA** only, respectively. During the whole therapeutic period, the body weight changes of the five groups mice showed an upward tendency and no damage was observed in the major organs (heart, liver, spleen, lung, and kidney) (Figs. 7*C* and S22). The high therapeutic biosafety of **DSF@MOF-199@FA** was further validated through blood routine examination (Fig. S23). Remarkably, compared with the other groups, **DSF@MOF-199@FA** group exhibited a significantly suppressed effect on 4T1 tumor growth (Figs. 7*D*, S24 and S25). Furthermore, the TUNEL, Ki-67, and H&E stained tumor pathological sections showed that the **DSF@MOF-199@FA** can induce cell necrosis in the tumor section, indicating the new anticancer drug formulations based on DSF has the better anticancer efficiency (Figs. 7*E* and S26).

## Conclusion

In summary, we have constructed a TME-active nanotheranostic platform, **DSF@MOF-199@FA**, for amplified oxidative stress–induced enhanced CuET-mediated chemotherapy. The results show that the released DSF and Cu(II) ions in acidic environment can form toxic CuET species *in situ* to induce chemotherapy outcome. Meanwhile, the therapeutic effect can be further enhanced by the copper ions mediated amplified oxidative stress through ∙OH generation (origin from Fenton-like reaction) and GSH consumption. This work not only represents a distinctive paradigm of a TME-activated nanosystem for amplified oxidative stress–induced enhanced CuET-mediated chemotherapy but also provides insight into repurposing FDA-approved drugs as versatile cancer therapeutics for effective cancer treatment.

## Experimental procedures

### Synthesis of defective MOF-199

Cu(NO_3_)_2_ aqueous solution (0.9 ml, 0.1 M), CTAB aqueous solution (9.6 ml, 0.1 M), and benzene-1,3,5-tricarboxylate triethylammonium salt aqueous solution (0.6 ml, 0.1 M) were added in the mixture of ethanol (15 ml) and deionized water (15 ml). Next, the aforementioned mixture was stirred vigorously at the room temperature (RT) for 10 min and then collected by centrifugation (5000 rpm, 1 min).

### Synthesis of DSF@MOF-199

DSF (20 mg) was dispersed in the acetone (10 ml) first and then **MOF-199** (20 mg) was added under continuous sonication. The aforementioned mixture was stirred at RT for 4 h. The light blue products were obtained by centrifugation, washing, and drying after the reaction finished.

### Synthesis of MOF-199@FA/DSF@MOF-199@FA

FA (50 mg) was dispersed into N,N-dimethylformamide (DMF) (50 ml) under sonication. After that, **MOF-199**/**DSF@MOF-199** (50 mg) was added into the aforementioned solutions and stirred at 30 °C overnight without light interference. Thereafter, the products were washed with DMF three times to remove the excess FA and then washed with ethanol three times again; the light blue product was preserved in ethanol.

### Synthesis of DSF@MOF-199@FA-FITC

FITC (2 mg) was dispersed into DMF (2 ml) and then **DSF@MOF-199@FA** (2 mg) was added into the aforementioned solutions and stirred overnight. Thereafter, the products were washed with DMF and ethanol three times, respectively. The light blue product was preserved in ethanol.

### Synthesis of CuET

About 87 mg DSF and 50 mg CuCl_2_ were added to 100 ml deionized water and stirred at RT for 24 h. The solution was extracted with chloroform and dried to form a black solid.

## Data availability

All data generated or analyzed during this study are included in this published article and its additional files.

## Supporting information

This article contains supporting information.

## Conflict of interest

The authors declare that they have no conflicts of interest with the contents of this article.
