# Supplementary material for: A tumor microenvironment-activated metal-organic framework–based nanoplatform for amplified oxidative stress–induced enhanced chemotherapy
Source: J Biol Chem. 2022 Nov 23;299(1):102742. doi: 10.1016/j.jbc.2022.102742 (PMC9793314; doi:10.1016/j.jbc.2022.102742)
Supplement: Supplemental Figs. S1–S26 [file mmc1.pdf]

Supporting Information

A Tumor Microenvironment-Activated Metal-Organic Framework-Based Nanoplatform for Amplified Oxidative Stress-Induced Enhanced Chemotherapy

Bo Li, Xin Yao, Jiaqi Li, Xin Lu, Wen Zhang, Wenyao Duan, Yupeng Tian, and Dandan Li[*]

Instrument. UV-vis absorption spectra were recorded on a UV-265 spectrophotometer. Fluorescence measurements were carried out on a Hitachi F-7000 fluorescence spectrophotometer. SEM and Mapping were detected by REGULUS8230*. TEM were carried on a JEM-2100. PXRD patterns were recorded on Smart Lab 9KW. Fluorescence measurements were carried out on a Hitachi F-7000 fluorescence spectrophotometer. IR spectra were recorded on a Nicolet FT-IR instrument (mid-IR: 4000 ~ 400 cm^{-1} range with KBr discs). Confocal laser scanning microscopy (CLSM) images were performed using Lecia TCS SP8 DIVE FALCON which equipped with single-wavelength laser (output wavelength: 405 nm, 456 nm, 488 nm, 514 nm, 561 nm, 633 nm).

Materials: All starting materials were obtained from commercial supplies and used without further purification. The chemicals of trimesic acid (98%), Hexadecyl trimethyl ammonium bromide (99%), triethylamine (99%), disulfide (99%), copper nitrate trihydrate (99.99%) and folic acid (99%) were purchased from Aladdin Co., Ltd. The 2′,7′-dichlorofluorescein diacetate (DCFH-DA) was purchased from Macklin Co., Ltd. (4,5-dimethylthiazol-2-yl)-2,5-diphenyltetrazolium bromide (MTT) was obtained from Beyotime Biotech Co., Ltd. (China). Annexin V-FITC/PI Apoptosis Detection Kit and Calcein AM /PI Kit was obtained from Shanghai Bestbio (China). Ethanol and N, N-Dimethylformamide (99.5%) were purchased from Sinopharm Chemical Reagent Co., Ltd. Deionized (DI) water was used throughout.

Hydroxyl radical (·OH) detection by Terephthalic acid (TA). Terephthalic acid (0.05 mmol) was dissolved into 100 mL NaOH solution (2 mM). Next, H_2O_2 (1 mM, 200 μL) and **DSF@MOF-199@FA** (1 mg mL^{-1}, 100 μL) were added into 1 mL PBS buffer solution and incubated for 5 minutes. 1 mL terephthalic acid was added into the above mixtures and incubated for another 5 minutes. The fluorescence of the mixture was measured at 435 nm in different time (λ_{ex} = 315 nm, λ_{em} = 420-450 nm).

GSH detection. Amplite® fluorimetric glutathione GSH/GSSG ratio assay kit was selected as the detection agent for GSH. 2 mL PBS buffer solution (pH = 7.4) was added with GSH (1 mM). And then, **DSF@MOF-199@FA** (50 μg mL^{-1}), **DSF@MOF-199@FA**+AA (25 μg mL^{-1}) and **DSF@MOF-199@FA**+H_2O_2 (100 μM) were added into the above solution incubated for 30 minutes. Thiolite™ Green (10 μL) was added into the above mixtures and incubated for another 30 minutes. The fluorescence spectra of the mixture were measured around 520 nm in different time.

Detection of the CuET production. The experiment of CuET production was performed at 37 °C in PBS solution (pH = 6.0). First, the as-prepared **DSF@MOF-199@FA** (2 mg mL^{-1}) was dispersed into PBS with different pH at room temperature. At varied time points, the absorption spectra of the solution were recorded.

Assessment of biodegradability of MOF-199 and DSF@MOF-199. **MOF-199** (4 mg) and **DSF@MOF-199** (4 mg) were immersed in PBS (4 mL) with different pH values (7.4, 6.5), and then these vials were placed into a water bath (37 °C) with slowly stirring (200 rpm) to mimic natural conditions. Subsequently, testing solution (2 mL) was sampled at different time interval (0, 0.5, 2 h) and the structural changes of **MOF-199** were observed using SEM. The UV-vis absorbance spectra of the mixture (with 2 h incubation) were measured around 300-600 nm.

Culture of 3D multicellular tumor spheroids (3D MCTs). 5 mL Poly HEMA solution was added to 25 mL cell culture flask, the ethanol was evaporated at 37 °C, and then sterilized under ultraviolet lamp for 3 - 5 h. The culture flask was washed twice with PBS, and then 1 mL liquor of 4T1 cells was added. When the cell mass density was relatively high, the flask treatment was conducted, and the cells were further cultured for 3 - 5 days, 3D multicellular spheroids could be formed with appropriate diameter. 3D MCTs were incubated with **DSF@MOF-199@FA** (50 μg mL^{-1}) for 12 h. Then, 3D MCTs stained with AM and PI for 15 min, then washed with PBS solution and analyzed by confocal laser scanning microscope (CLSM).

Cell uptake analysis. HEK 293T cells (FAR-negative cells), HeLa cells (FAR-positive cells) 4T1 cells (FAR-positive cells) were seeded onto corresponding cell culture dishes and grown to about 70% confluency before used. HEK 293T cells, HeLa cells, 4T1 cells, HeLa cells (treated with free FA 1 h in advance) and 4T1 cells (treated with free FA 1 h in advance) were treated with **DSF@MOF-199@FA-FITC** (50 μg mL^{-1}), respectively. And after 4 h incubation, the cellular uptake ability of **DSF@MOF-199@FA-FITC** were analyzed using CLSM.

Cytotoxicity assays in cells. The study of the enhanced chemotherapeutic effect of **DSF@MOF-199@FA** was carried out using the methylthiazolyldiphenyltetrazolium bromide (MTT) assay. **DSF@MOF-199@FA** stock solutions were diluted by fresh medium in to desired concentration (0, 10, 20, 40, 80 μg mL^{-1}). 4T1 cells or HEK 293T cells were cultured in a 96-well plate for 24 h before experiments. The cell medium was then exchanged by different concentrations of **DSF@MOF-199@FA** medium solutions. They were incubated at 37 °C in 5% CO_2 for 12 h. The cell medium solutions were exchanged by 100 μL of fresh medium, followed by the addition of 20 μL (5 mg mL^{-1}) MTT solution to each well. The cell plates were then incubated at 37 °C in 5% CO_2 for 4 h. After MTT medium removal, the formazan crystals were dissolved in DMSO (100 μL $well^{-1}$) and the absorbance was measured

at 490 nm using a microplate reader. And duplicated experiments have been tested.

Intracellular ROS detection. The intracellular ·OH was measured using the fluorescent probe hydroxyphenyl fluorescein (HPF). 4T1 cells were incubated with 50 μg mL^{-1} **DSF@MOF-199@FA** with/without H_2O_2 for 4 hours, and HPF was added following. After that, confocal fluorescence imaging was performed to give the level of intracellular ·OH with the excitation wavelength of 488 nm and emission wavelength from 500 nm to 520 nm.

Intracellular GSH detection. To detect the depletion of GSH, the 4T1 cells were seeded into 6-well plate for 24 h before experiments. Then, the **DSF@MOF-199@FA** (50 μg mL^{-1}), AA (25 μg mL^{-1}) and H_2O_2 (100 μM) were added and incubated for 24 h. After that, the cells were washed with PBS (three times) and treated with trypsin (0.25%) to collect 4T1 cells. Then, the 4T1 cells were resuspended in 1 mL of PBS and handled by an ultrasound cell crusher. After that, 0.1 mL of the above 4T1 cell suspensions were mixed with 5 μL of thiolite green for 5 min. The depletion of intracellular GSH was measured by fluorescence spectroscopy at 520 nm.

Flow Cytometry Study. Cells seeded into the 6-well plates were cultured for 24 h. Next, the medium was replaced with medium (3 mL) containing **DSF@MOF-199@FA** (50 μg mL^{-1}), AA (25 μg mL^{-1}) and H_2O_2 (100 μM), at 37 °C for 12 hours. The cells were collected by centrifugation and resuspended in binding buffer containing Propidium Iodide (PI, 10 μL) and Annexin-V FITC (5 μL) for 15 min. The signal was collected by a BD FACS Calibur flow cytometer (Beckaman/Gallios).

Live/Dead assay with calcein AM/PI. 4T1 cells with a density of 10^5 cells per well were cultured in a 6-well plate for 24 h to allow the attachment of cells. After cells were washed twice by PBS solution, **DSF@MOF-199@FA** (50 μg mL^{-1}), AA (25 μg mL^{-1}) and H_2O_2 (100 μM) were added to above culture medium. The cells were incubated for 12 h, calcein AM and

PI were used to confirm the viability of 4T1 cells. Fluorescence images were collected by CLSM.

In Vivo Blood Circulation Test of DSF@MOF-199@FA. To evaluate circulation time in the bloodstream, blood samples (20 μL) were taken from female BALB/C mices (n = 5) at various time points (5 min, 10 min, 0.5, 1, 2, 4, 6, 8, 12 and 24 h) after intravenous injection of **DSF@MOF-199@FA** in a saline solution (100 μL, 4 mg mL^{-1}). Cu concentration in the obtained blood samples (melted by chloroazotic acid) were measured by ICP-OES.

In Vivo Tumor Chemotherapy. All the animal procedures were approved by the Institutional Animal Care and Use Committee of Anhui University (serial number: 2020-042) based on the National Standard of China GB/T35892-2018 guidelines for Ethical Review of Experimental Animal Welfare. To evaluate the in vivo therapeutic effect of **DSF@MOF-199@FA**, female BALB/C mice were randomly divided into five groups (n = 5) and then 4T1 cells (1×10^6 cells per mouse) were injected subcutaneously into their chest to establish transplanted tumor models. When the tumors which located above the chest cavity grew to 80 mm^3, PBS, DSF, **MOF-199**, **DSF@MOF-199** and **DSF@MOF-199@FA** were administered by injection via the tail vein (3.75 mg kg^{-1}). Body weight and tumor volume were measured every other day after chemotherapeutic administration. All the tumors were then sectioned into slices, and TUNEL, antigen Ki-67 and H&E staining was performed for histological analysis. The treated mice were sacrificed and their organs (heart, liver, lung, spleen, and kidney) and blood samples were harvested for further analysis, which included blood-index monitoring and H&E staining.

Statistical Analysis. Statistical analyses were carried out by Student's t-test. All the experiment Data were expressed as means ± SD. The differences of all date were regarded as significant for p-value: n.s., not significant; * $P < 0.05$, ** $P < 0.01$, *** $P < 0.001$.

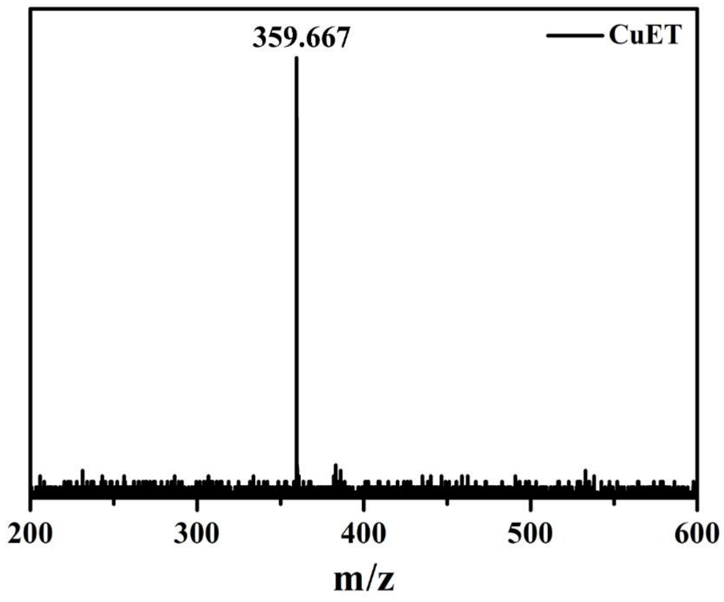

Fig. S1 Mass spectrum of CuET.

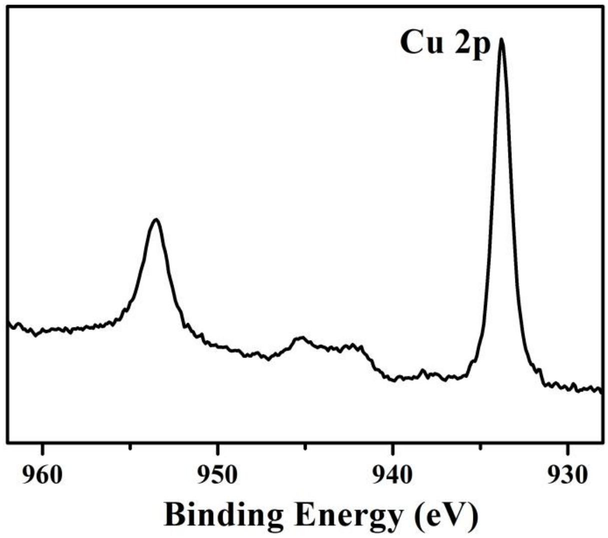

Fig. S2 XPS spectrum of Cu 2p in CuET.

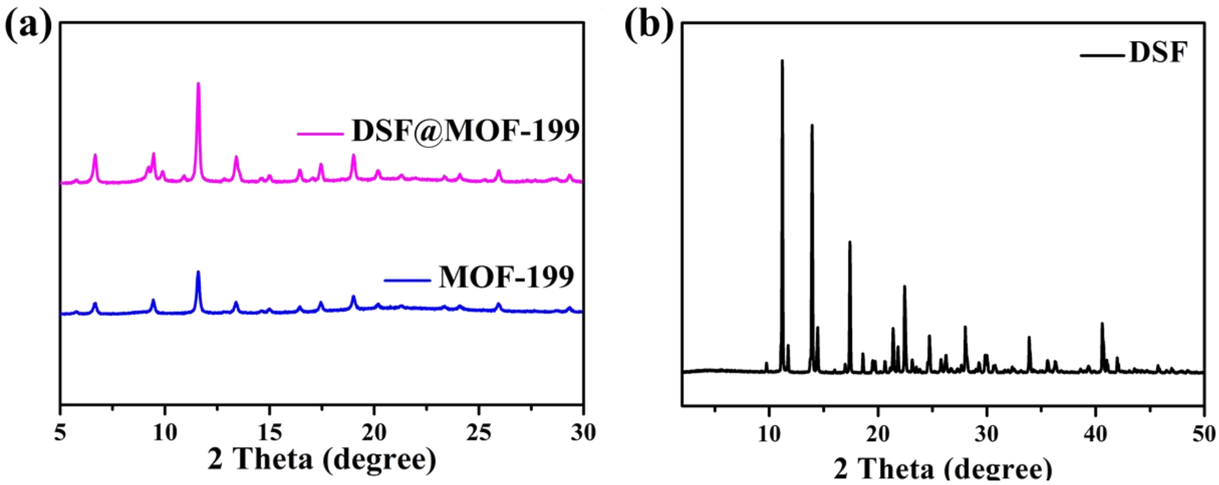

Fig. S3 (a) PXRD patterns of **MOF-199** and **DSF@MOF-199**. (b) PXRD patterns of DSF. The XRD results show that that the two new diffraction peaks (2θ = 9.25°, 9.95°) of **DSF@MOF-199** were not consistent with the characteristic peaks of DSF.

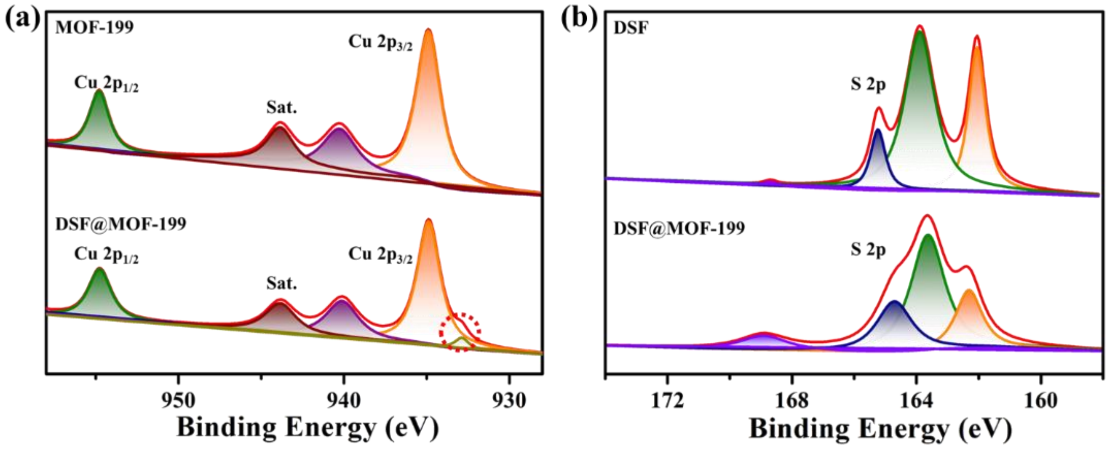

Fig. S4 (a) XPS spectra analysis of Cu 2p orbit of **MOF-199** and **DSF@MOF-199**. (b) XPS spectra analysis of S 2p orbit of **DSF** and **DSF@MOF-199**. The obvious changes of the Cu 2p and S 2p spectra for **DSF@MOF-199** compared with the Cu 2p spectrum of **MOF-199** and the S 2p spectrum of DSF implied the interaction between DSF molecules and Cu ions of MOFs.

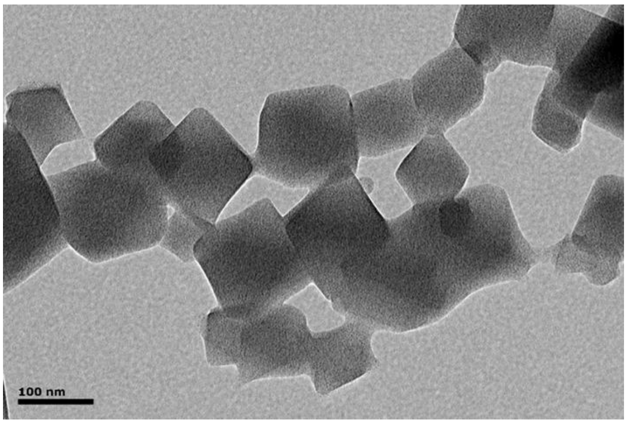

Fig. S5 TEM image of **MOF-199** (scale bar: 100 nm).

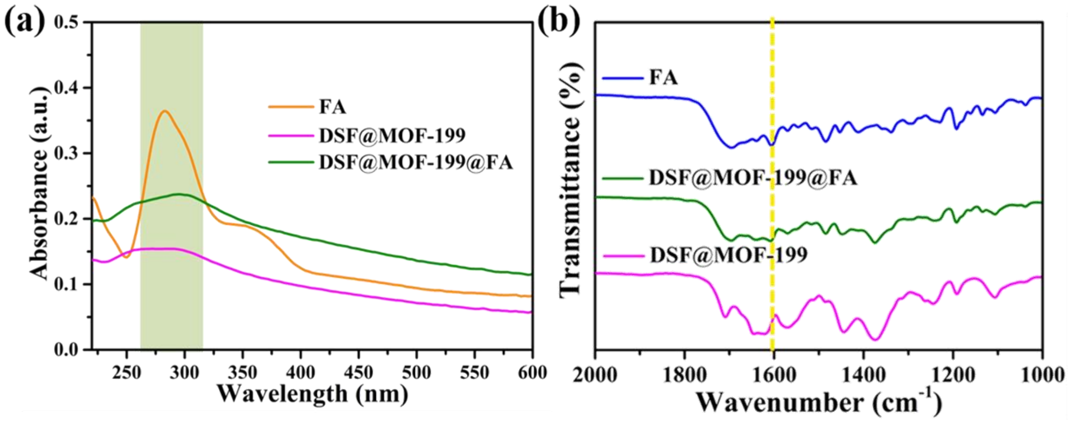

Fig. S6 (a)UV-vis, and (b)FTIR of FA, **DSF@MOF-199** and **DSF@MOF-199@FA**.

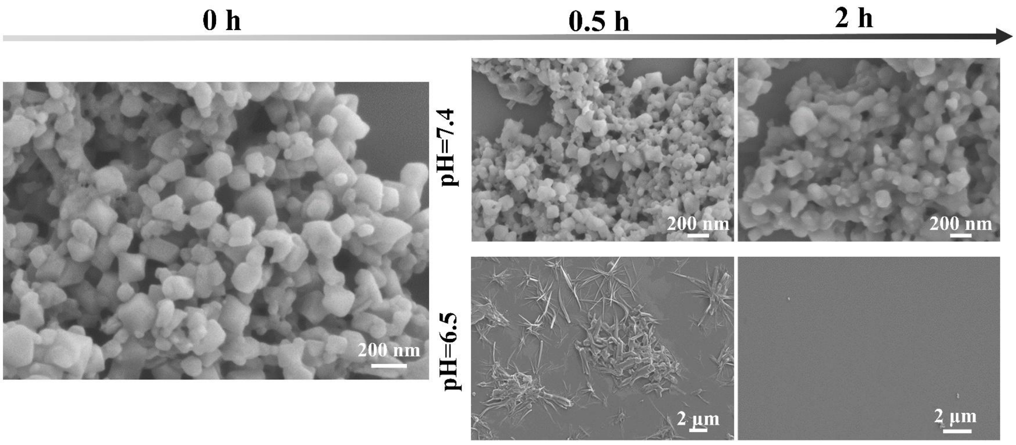

Fig. S7 SEM images of **MOF-199** incubated in neutral or acidic environment within 2 hours.

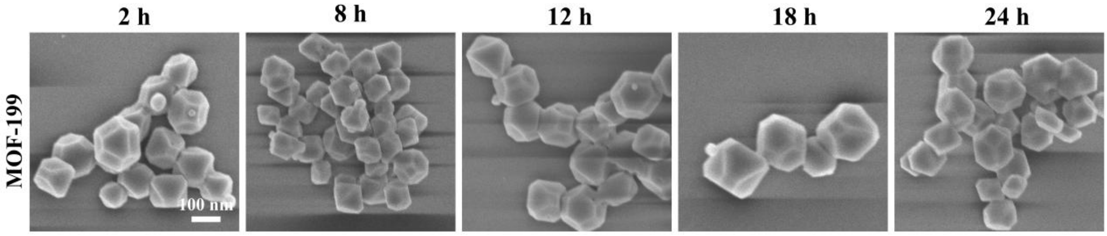

Fig. S8 The morphology of **MOF-199** in deionized water solution at different time.

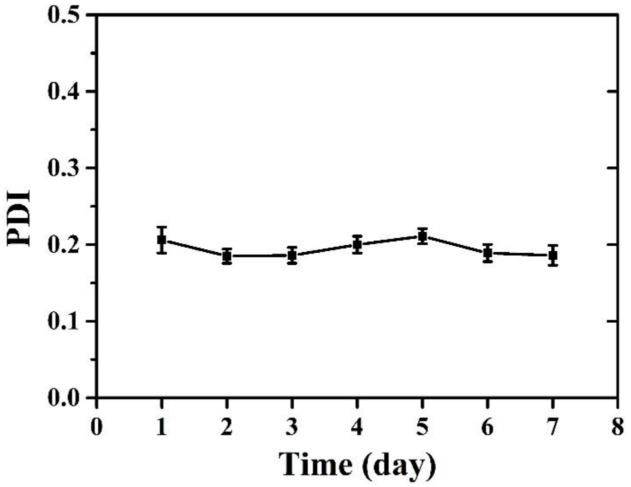

Fig. S9 PDI changes of **DSF@MOF-199@FA** for 7 days in serum.

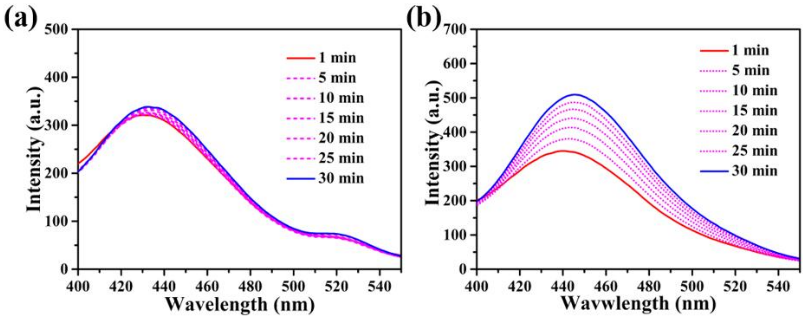

Fig. S10 Determination of the formation of ·OH by terephthalic acid as a fluorescent probe. (a) pH = 7.4. (b) pH = 6.5.

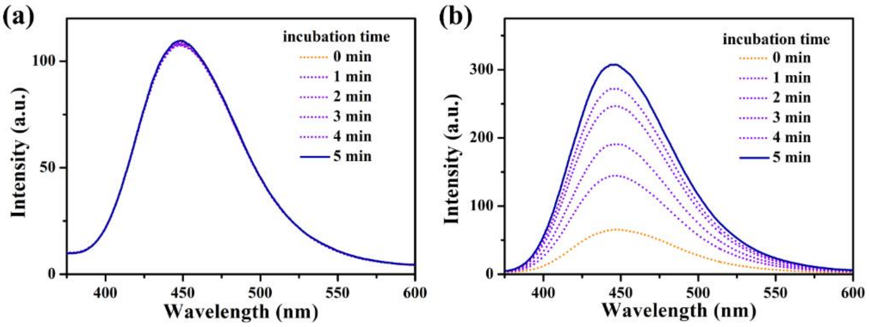

Fig. S11 Determination of the formation of ·OH by terephthalic acid as a fluorescent probe. (a) **CuET** (10 μM). (b) $CuCl_2$ (10 μM).

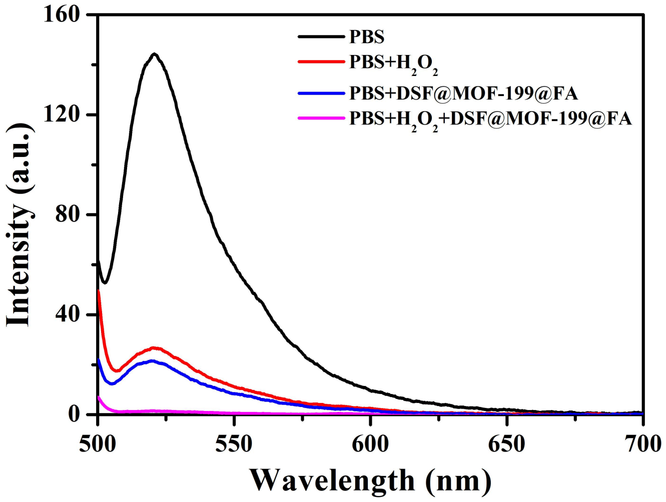

Fig. S12 GSH depleting ability of different samples. Thiolite™ Green was chosen as the detection agent for GSH.

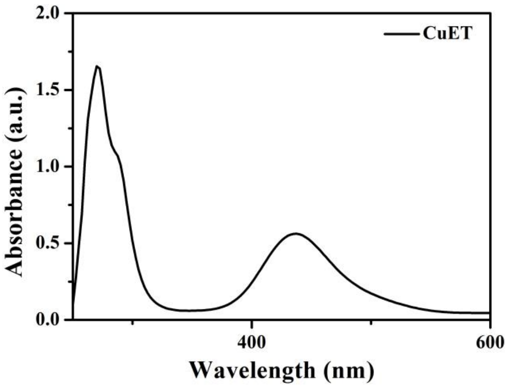

Fig. S13 Absorption spectra of **CuET**.

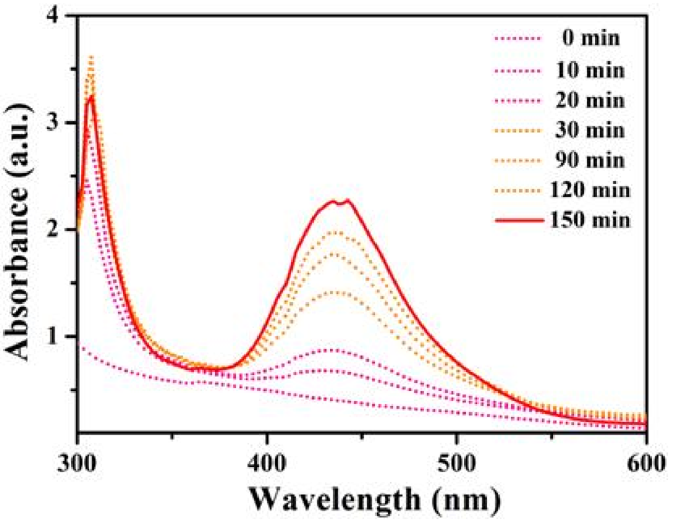

Fig. S14 Determination of the formation of CuET (**DSF@MOF-199@FA**: 2 mg/mL, pH = 6.5).

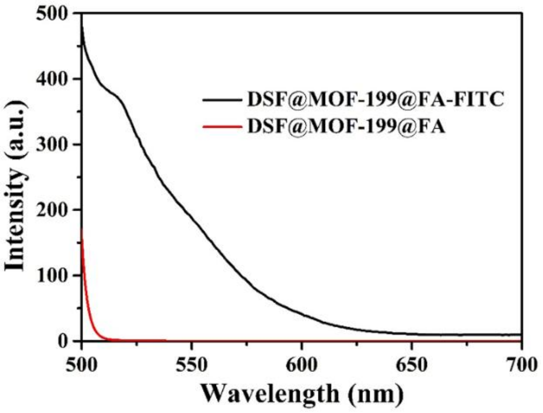

Fig. S15 Emission spectra of **DSF@MOF-199@FA** and **DSF@MOF-199@FA-FITC** (λ_{ex} = 490 nm).

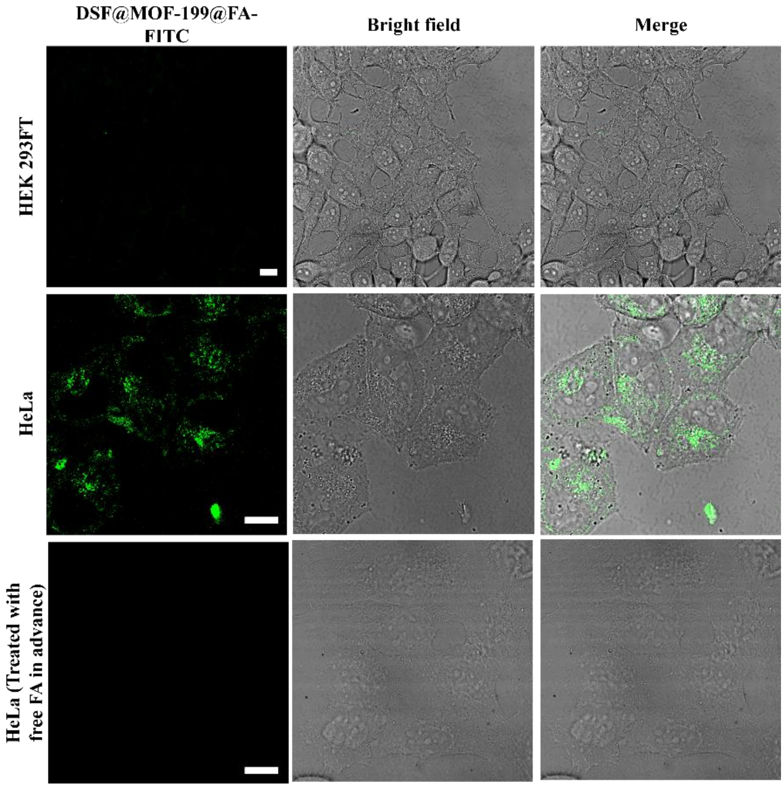

Fig. S16 Confocal images to check the cell uptake of **DSF@MOF-199@FA-FITC** (scale bar: 20 μm).

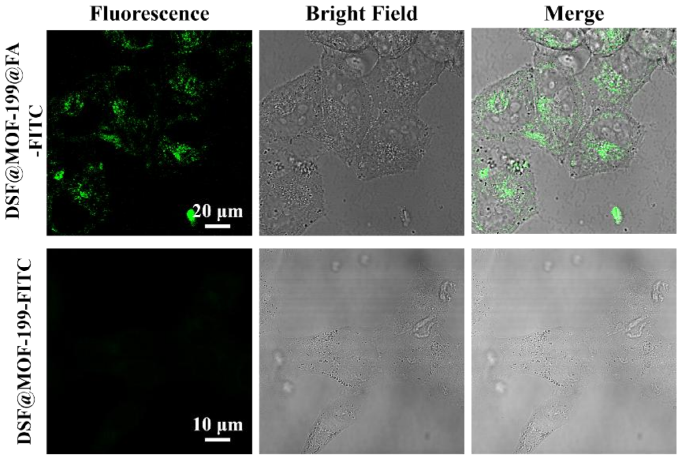

Fig. S17 Confocal images to check the Hela cells uptake of **DSF@MOF-199 -FITC**. The confocal images of **DSF@MOF-199@FA-FITC** were derived from Fig.S16 to highlight the remarkable change of fluorescence and demonstrate the modification of FA could make **DSF@MOF-199** target Hela cells.

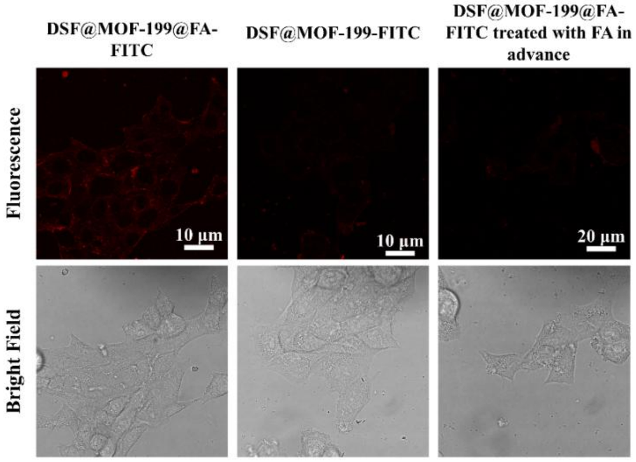

Fig. S18 Confocal images to check the 4T1 cell uptake of **DSF@MOF-199@FA-FITC**.

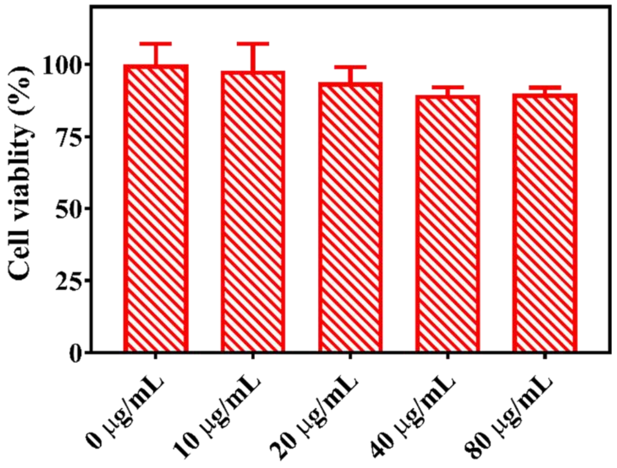

Fig. S19 Cell viability in HEK 293T cells after 12 h of incubation with different concentration of **DSF@MOF-199@FA**.

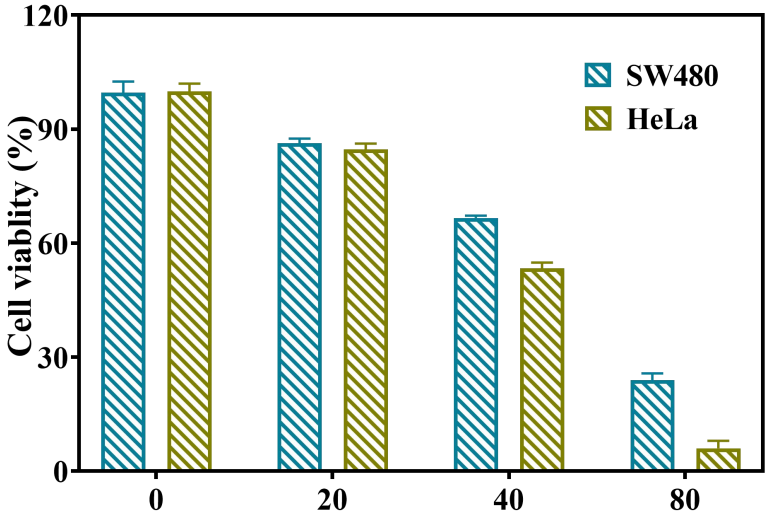

Fig. S20 Cell viability of the **DSF@MOF-199@FA** nanoparticles in HeLa and SW480 cancer cells after 12 h of incubation with different treatments.

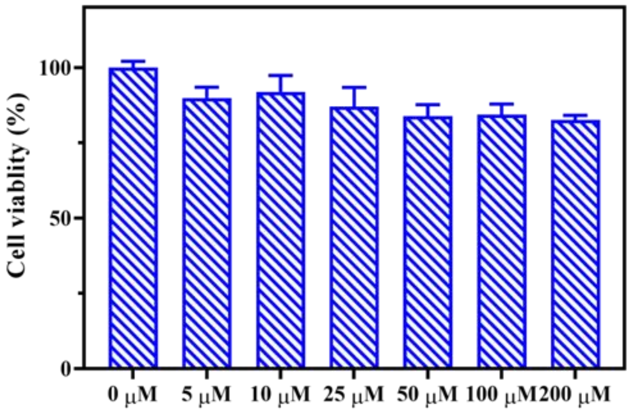

Fig. S21 Cell viability in 4T1 cells after 12 h of incubation with different concentration of H_2O_2.

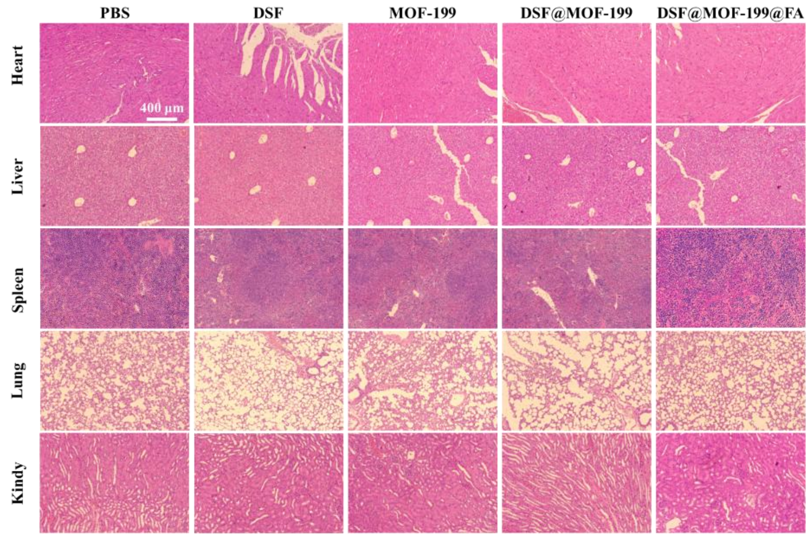

Fig. S22 H&E staining histological sections of major organs of nude mice bearing 4T1 tumor of different groups after varied treatments (PBS, DSF, **MOF-199**, **DSF@MOF-199** and **DSF@MOF-199@FA**).

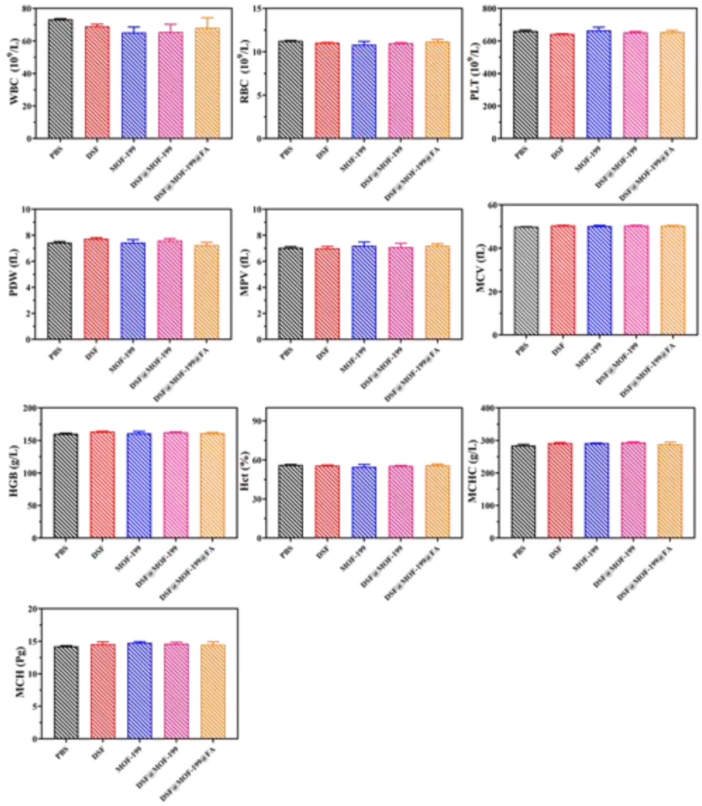

Fig. S23 Biosafety analysis. The data were achieved from mice treated with PBS, DSF, **MOF-199**, **DSF@MOF-199** and **DSF@MOF-199@FA** at 14 days. Hematology data containing WBC, RBC, PLT, PDW, MPV, MCV, MCHC, MCH, HGB and HCT.

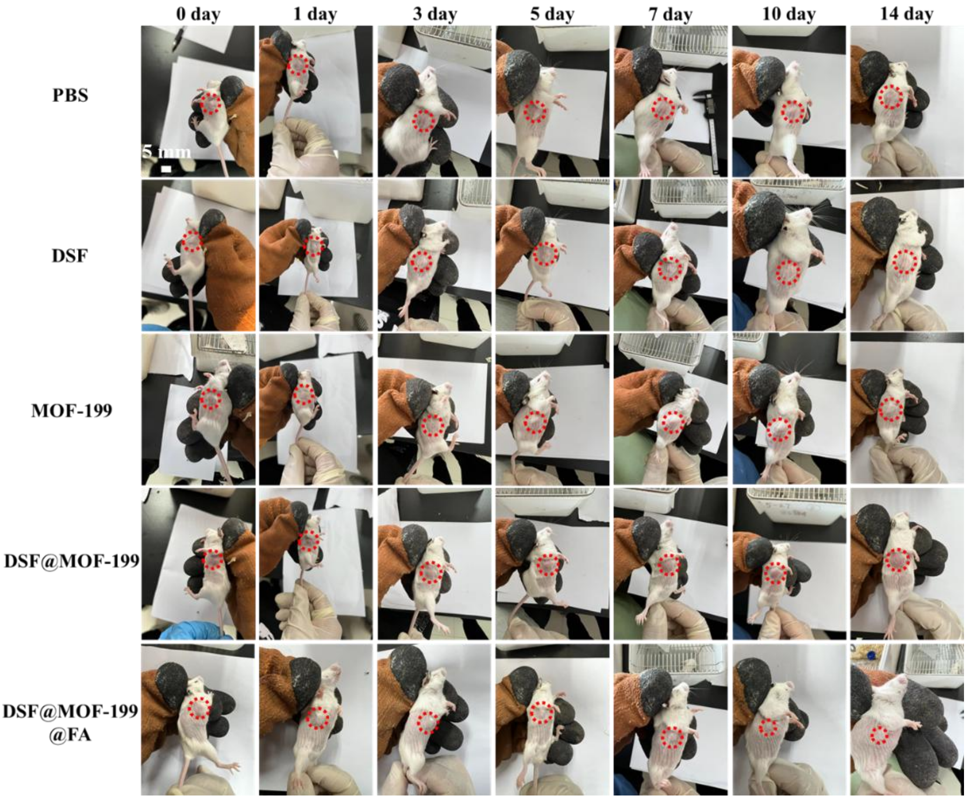

Fig. S24 Digital images of mice from the PBS, DSF, **MOF-199**, **DSF@MOF-199** and **DSF@MOF-199@FA** groups wherein tumor growths were compared at the beginning and end of treatment.

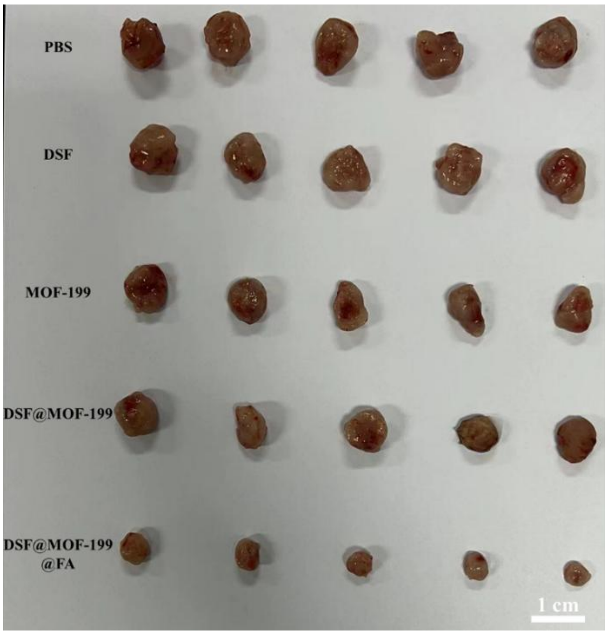

Fig. S25 Digital photographs of excised tumors after 14 days of various treatments.

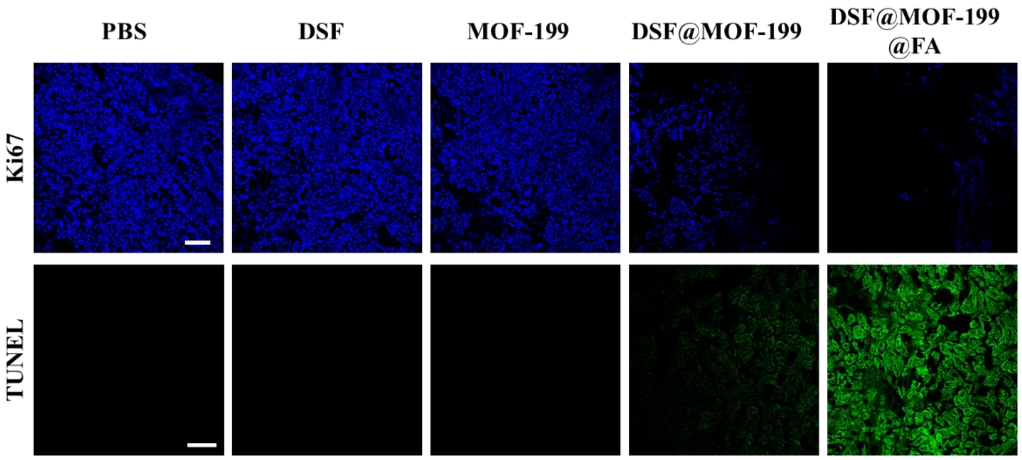

Fig. S26 TUNEL, antigen Ki-67 immunofluorescence-stained sections of 4T1 tumors in different treatment groups. Scale bar: 100 μm.
